# Endovascular treatment for massive haemoptysis due to pulmonary pseudoaneurysm: report of 23 cases

**DOI:** 10.1186/s13019-023-02346-7

**Published:** 2023-08-14

**Authors:** Fen-Qiang Li, Dong-Jun Su, Wan-Jia Zhang, Zhong-Ke Chen, Geng-Xiang Li, Shuang-Xi Li, Yu-xing Peng, Lei Dang, Wen-Hui Wang

**Affiliations:** 1 Department of Interventional Radiology, The Frist Hospital of Lanzhou University, Lanzhou, China; 2 Department of Vascular and Tumour Intervention, Liangzhou Hospital, Wuwei City, Gansu Province China; 3 Department of Interventional Radiology, Affiliated Hospital of Gansu Medical College, PingLiang, Gansu Province China

**Keywords:** Pulmonary artery pseudoaneurysms, Haemoptysis, Endovascular treatment

## Abstract

**Purpose:**

To evaluate the safety and effectiveness of endovascular treatment for massive haemoptysis caused by pulmonary pseudoaneurysm (PAP).

**Methods:**

The clinical data, imaging data, and endovascular treatment protocol of 23 patients with massive haemoptysis caused by continuous PAP were retrospectively analysed. The success, complications, postoperative recurrence rate, and influence of the treatment on pulmonary artery pressure were also evaluated.

**Results:**

Nineteen patients with a bronchial artery-pulmonary artery (BA-PA) and/or nonbronchial systemic artery-pulmonary artery (NBSA-PA) fistula underwent bronchial artery embolization (BAE) and/or nonbronchial systemic artery embolization (NBSAE) + pulmonary artery embolization (PAE). The pulmonary artery (PA) pressures before and after embolization were 52.11 ± 2.12 (35–69 cmH_2_O) and 33.58 ± 1.63 (22–44 cmH_2_O), respectively (P = 0.001). Four patients did not have a BA-PA and/or NBSA-PA fistula. Embolization was performed in two patients with a distal PAP of the pulmonalis lobar arteria. Bare stent-assisted microcoils embolization was performed in the other two patients with a PAP of the main pulmonary lobar arteries. The PA pressures of the four patients before and after treatment were 24.50 ± 1.32 (22–28 cmH_2_O) and 24.75 ± 1.70 (22–29 cmH_2_O), respectively (P = 0.850). The technique had a 100% success rate with no serious complications and a postoperative recurrence rate of 30%.

**Conclusion:**

Endovascular treatment is safe and effective for massive haemoptysis caused by PAP. BAE and/or NBSAE can effectively reduce pulmonary hypertension in patients with a BA-PA and/or NBSA-PA fistula.

## Introduction

Massive haemoptysis is defined as the expectoration of greater than 500ml of blood per day and has a mortality rate of more than 50%. Most patients have bronchial artery (BA) and/or nonbronchial systemic artery (NBSA)-related haemoptysis, with only 5-10% of patients having pulmonary artery (PA)-related haemoptysis [1].

Haemoptysis is also caused by pulmonary artery pseudoaneurysms (PAPs), which have diverse aetiologies, including trauma, iatrogenesis (percutaneous lung biopsy or tumour ablation), infection (tuberculosis, aspergillosis), and tumours [2]. The 1-year mortality rate of patients with a PAP is approximately 50–100%, and the main cause of PAP-related deaths is asphyxia caused by rapid and massive intrapulmonary haemorrhage [3, 4]. Therefore, early diagnosis and treatment are crucial for the prognosis and survival of PAP patients.

In 1960, pneumonectomy was reported to reduce mortality by up to 15% for PAP patients. Due to the high risk of surgery and the fact that some patients cannot tolerate surgery, BAE or NBSAE is suggested as first-line measures in most studies. Research also shows that 38% of patients who have successfully experienced BAE or NBSAE will have haemoptysis again [5]. There are only few literature reports on the management of PAPs through the PA pathway during simultaneous BAE and NBSAE if there are BA-PA or NBSA-PA fistulas. The main purposes of this study were to retrospectively analyse the outcomes of 23 PAP patients with massive haemoptysis, explore the safety and efficacy of PAPE and BAE and/or NBSAE for such patients, and provide feasible treatment methods for massive haemoptysis caused by pulmonary pseudoaneurysm.

## Patients and methods

This was a three-centre, retrospective cohort analysis of 23 consecutive patients who were initially diagnosed as having a PAP and were treated with endovascular management between July 2014 and January 2022. Inclusion criteria: ①Diagnosis of PAP by enhanced CT ②Complete follow-up data ③Signed informed consent form before surgery. Exclusion criteria: ①Incomplete follow-up data ② Lost to follow-up. Of the 23 patients, 22 had a clear history of tuberculosis, and 1 had an unknown aetiology and was diagnosed as having vasculitis by the multidisciplinary team (MDT). The study was approved by the Ethics Committee of the First Affiliated Hospital of Lanzhou University, China.

### Computed tomography angiography (CTA)

Spiral CT scans were performed using a helical CT machine above 64 slices at 5–10 cm above the thoracic inlet to the upper abdomen to prevent omitting the blood vessels responsible for causing haemoptysis. Thin layer images with a thickness and spacing of 1.00 mm were reconstructed after scanning. CTA examination was performed before angiography to detect PAPs.

### Treatment methods

All interventional operations were performed by associate chief physicians or a physician with a higher title. All patients with a BA-PA or/and NBSA-PA fistula underwent BAE + NBSAE via the femoral artery. An appropriate 5-F catheter (COBRA Terumo Japan, YASHIRO Terumo Japan, MIK Cook USA) was selected based on the results of the chest enhanced CT scan. The target vessels, including the tortuous dilated BA and/or NBSA, with or without a BA-PA and/or NBSA-PA fistula were then identified [6]. The catheter was temporarily retained if PAP development was detected in the BA and/or NBSA, and BAE and/or NBSAE was performed after PAPE via PA. However, BAE and/or NBSAE was performed when PAP development was not detected. The pigtail catheter was placed in the main PA, and the PA pressure was measured before and after endovascular therapy. The influence of a BA-PA or NBSA-PA fistula and target vessel embolization on PA pressure was evaluated according to the change in pressure.

#### PAPE method for distal pulmonalis lobar arteria

The right femoral vein was punctured percutaneously by using the Seldinger technique. A 5 F pigtail catheter (Terumo Japan) was introduced into the left and right pulmonary artery trunk under angiography of the right atrium and ventricle guided by 0.035 guidewires (Terumo Japan). The surgeon chose the most appropriate 5-F catheter (COBRA Terumo Japan, Vertebral Terumo Japan) and then inserted it into the PAP offending artery or the PAP with a 0.035 exchange guide wire (260 cm Terumo Japan). The location and size of the PAP were further determined via manual angiography. A microcatheter (Stride Asahi Japan, Progreat Terumo Japan) was crossed over the PAP as far as possible and introduced into the distal end of the pulmonalis lobar arteria before PAP embolization at the distal end of the pulmonalis lobar arteria. The position of the microcatheter was determined using manual angiography. Interlock microcoils (Fibred CTM Occlusion System, Boston Scientific, USA) were introduced via fluoroscopic guidance for complete embolization of the PA branch and cavity.

#### PAPE of the main pulmonary lobar arteries

The 6 F long sheaths (SVA6F-900 China Prior health) were introduced through the bilateral femoral vein approach. A 5-F contrast catheter (COBRA Terumo Japan) and coaxial microcatheter were introduced into the PAP for cavity embolization. A stiff guide wire was introduced to one side of the responsible PA to further prepare for the release of the bare vascular stent (Cordis S.M.A.R. T CONTROL Nitinol stent system in the United States). The vascular stent was successfully released after accurate introduction of a microcatheter, and the stent completely covered the PAP neck. Interlock microcoils (Fibred CTM Occlusion System, Boston Scientific, USA) were introduced through a microcatheter reserved in the PAP cavity for embolization. The PAP was completely embolized and no contrast agent remained in the PAP cavity, indicating the completion of embolization [6–8].

#### BAE/NBSAE method

A microcatheter (Stride Asahi Japan, Progreat Terumo Japan) was introduced as far as possible into the responsible vessels via a 5 Fr catheter to avoid making contact with nonresponsible vessels and to further support diagnosis through microcatheter angiography. BAE and/or NBSAE were performed using polyvinyl alcohol particles (PVA; 350–710 μm, Hangzhou Alicon), a gelatine sponge (Nanjing Jinling), and gelatine sponge particles (710–1000 μm, Hangzhou Alicon). The contrast agent flowed into the responsible artery for 3–5 min, and then embolization was considered to be completed when the contrast agent was stagnated in the responsible artery [7, 9].

Technical success was defined as clear entry into an abnormal BA and/or NBSA and complete embolization of the PAP. Clinical success was defined as complete stop of haemoptysis after successful endovascular treatment during hospitalization. Clinical failure was defined as persistent haemoptysis after endovascular treatment with no reduction in haemoptysis volume, haemoptysis that only stopped or decreased for a short period, or massive haemoptysis that recurs during hospitalization [3, 5–8].

### Statistical analyses

SPSS 25.0 (SPSS Inc. ; Chicago, IL, USA) was used for all analyses. Quantitative results were expressed as means ± standard deviations, and qualitative results were expressed as a number (%).

## Results

### Baseline information

The baseline characteristics of the 23 patients, including age, sex, haemoptysis volume, follow-up time (month), NBSA, and underlying aetiology, are shown in Table 1. The PAP location is described in detail in Table 2. PAP diameter, BA-PA and/or NBSA-PA fistula, treatment method, embolization materials and changes in PA pressures before and after embolization are shown in detail in Table 3.

**Table 1:** Baseline characteristics of 23 patients

Characteristic	Value
Age,(years)	49.8 ± 12.6
Gender	
Male	13(56.5%)
Female	10(43.5%)
Hemoptysis volume (mm/24 h)	642.2 ± 176.1
Follow up time (months)	19.3 ± 7.6
NBSA	
Internal thoracic artery	10
External thoracic artery	3
Intercostal arteries	5
Thyrocervical trunk	12
Etiology Tuberculosis Non-tuberculosis	22(95.7%) 1 (4.3%)

**Table 2:** PAP location

PAP location	Value
Main pulmonalis lobar arteria of lung	2
Distal of main pulmonalis lobar arteria of lung	
Double upper lobe	2
Right superior lobe	8
Right middle lobe	1
Right inferior lobe	2
Left upper lobe	7
Left lower lobe	1

**Table 3:** PAP diameter, BA and/or NBSA-PA fistula, treatment method, embolization materials and changes of pulmonary artery pressure before and after embolization

Characteristic	Value
PAP diameter (mm) BA-PA and/or NBSA-PA fistula	10.7 ± 5.4 19
Treatment method	
BAE + NBSAE + PAPE	18
BAE + PAPE	1
Stent + PAPE	2
PAPE	2
Embolization materials	
Bare stent + Interlock micro coils Interlock micro coils	2 2
PVA + Interlock micro coils PVA + gelatin sponge particles + Interlock micro coils PVA + homemade gelatin sponge strips + Interlock micro coils	14 4 1
The pulmonary artery pressure (cmH_2_O) Exist BA-PA and/or NBSA-PA fistula	19
Before embolization	52.11 ± 2.12
After embolization	33.58 ± 1.63
No BA-PA and/or NBSA-PA fistula	4
Before embolization	24.50 ± 1.32
After embolization	24.75 ± 1.70

### Technical success rate

**Fig. 1:**
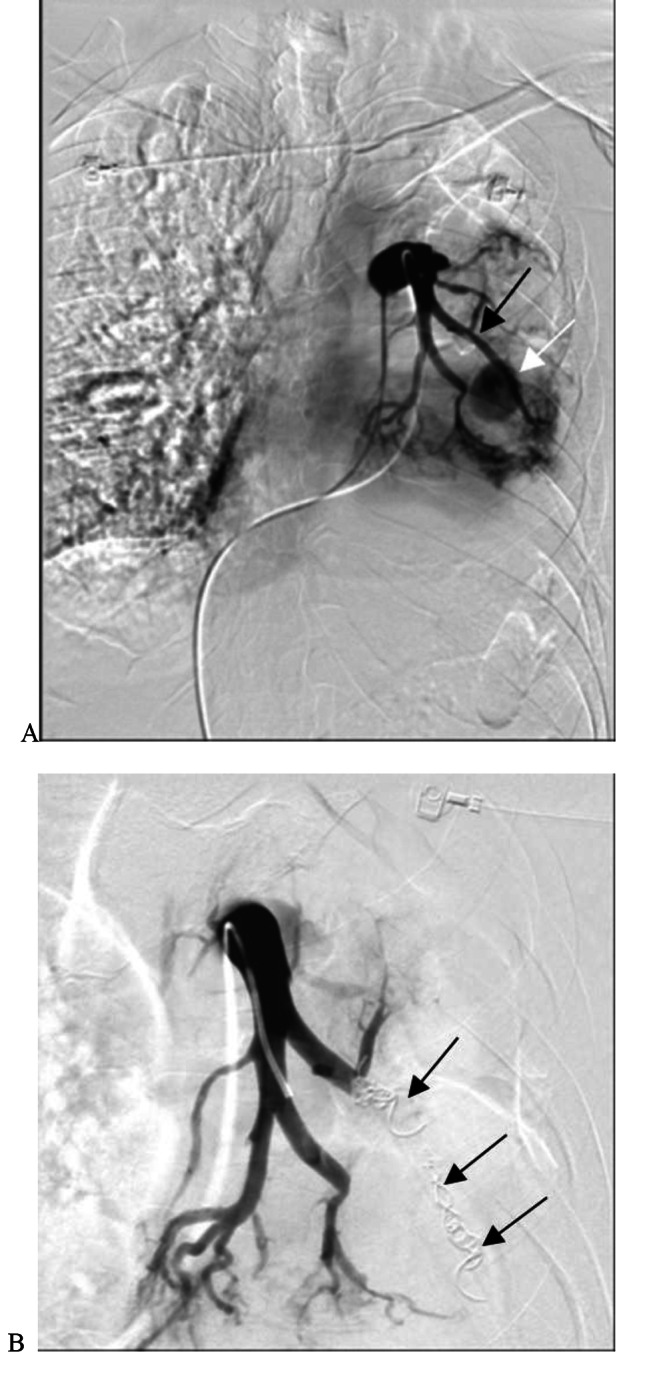
Sudden massive hemoptysis (about 600ml) of unknown cause. Contrast-enhanced CT showed a pseudoaneurysm of the left inferior pulmonary artery. **A**. Left pulmonary artery angiography shows the left inferior pulmonary artery (black arrow) was PAP (white arrow). **B**. The distal pulmonary artery (white arrow) of the left lower lobe of the lung was superselected with a microcatheter. Complete embolization was performed from the distal end to the proximal end with interlock coils (black arrow)

**Fig. 2:**
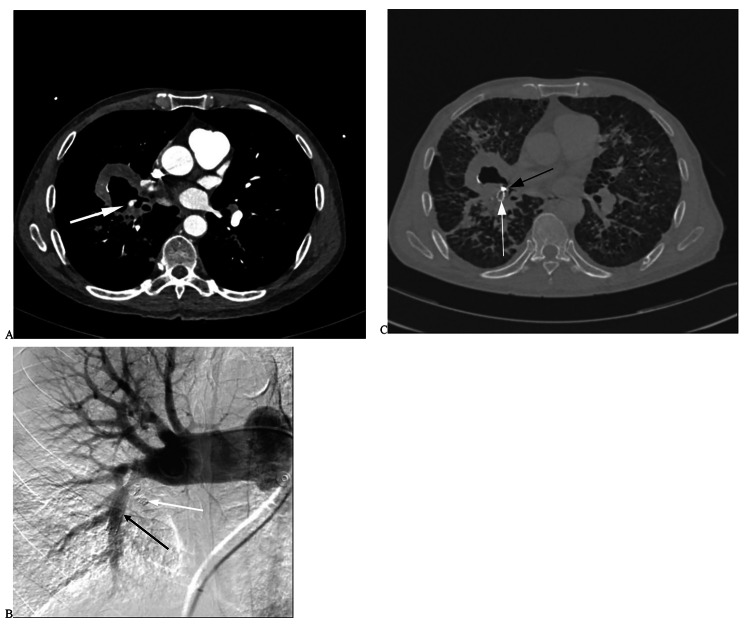
A patient with old tuberculosis was hospitalized with sudden massive hemoptysis for 1 day, and the volume of one-time hemoptysis was about 500ml. **A**. Chest enhanced CT shows cavity formation in the lower lobe of the right lung and PAP formation in pulmonalis lobar arteria (white arrow). **B**. No PAP was found after the stent (black arrow) assisted PAPE with a micros coils (white arrow) for the right lower pulmonalis lobar arteria. **C**. Postoperative chest CT scan shows the position of the stent (white arrow) and coils (black arrow)

Nineteen patients with a BA-PA and/or NBSA-PA fistula successfully underwent BAE and/or NBSAE + PAPE. BAE embolization was performed using polyvinyl alcohol particles (PVA; 350–710 μm, Hangzhou Alicon). Fourteen patients successfully underwent NBSAE of the branch vessels using PVA (350–710 μm). However, 3 cases in the internal thoracic artery, 1 case in the intercostal artery, and 1 case in the thyroid neck trunk were not only affecting the distal branch vessels; thus, a microcatheter was used. Protective embolization of the distal normal arterial trunk was performed using homemade gelatine sponge strips, and then embolization of the responsible arteries was performed using PVA. Two patients (8.70%) with a PAP of the distal pulmonalis lobar arteria successfully underwent PAPE (Fig. 1). The other two patients (8.70%) underwent stent-assisted PAPE of a PAP in the main pulmonary lobar arteries (Fig. 2). In this study, the technical success rate was 100%.

### Changes in pulmonary artery pressure

The PA pressures of patients with a BA and/or NBSA-PA fistula and patients without a fistula before and after endovascular therapy are shown in Table 2. The PA pressures of 19 patients with a BA and/or NBSA-PA fistula before and after embolization were 52.11 ± 2.12 (35–69 cmH_2_O) and 33.58 ± 1.63 (22–44 cmH_2_O), respectively (P = 0.001). The PA pressures of four patients without a BA and/or NBSA-PA fistula before and after the operation were 24.50 ± 1.32 (22–28 cmH2O) and 24.75 ± 1.70 (22–29 cmH_2_O), respectively (P = 0.850).

### Follow-up and complications

**Fig. 3:**
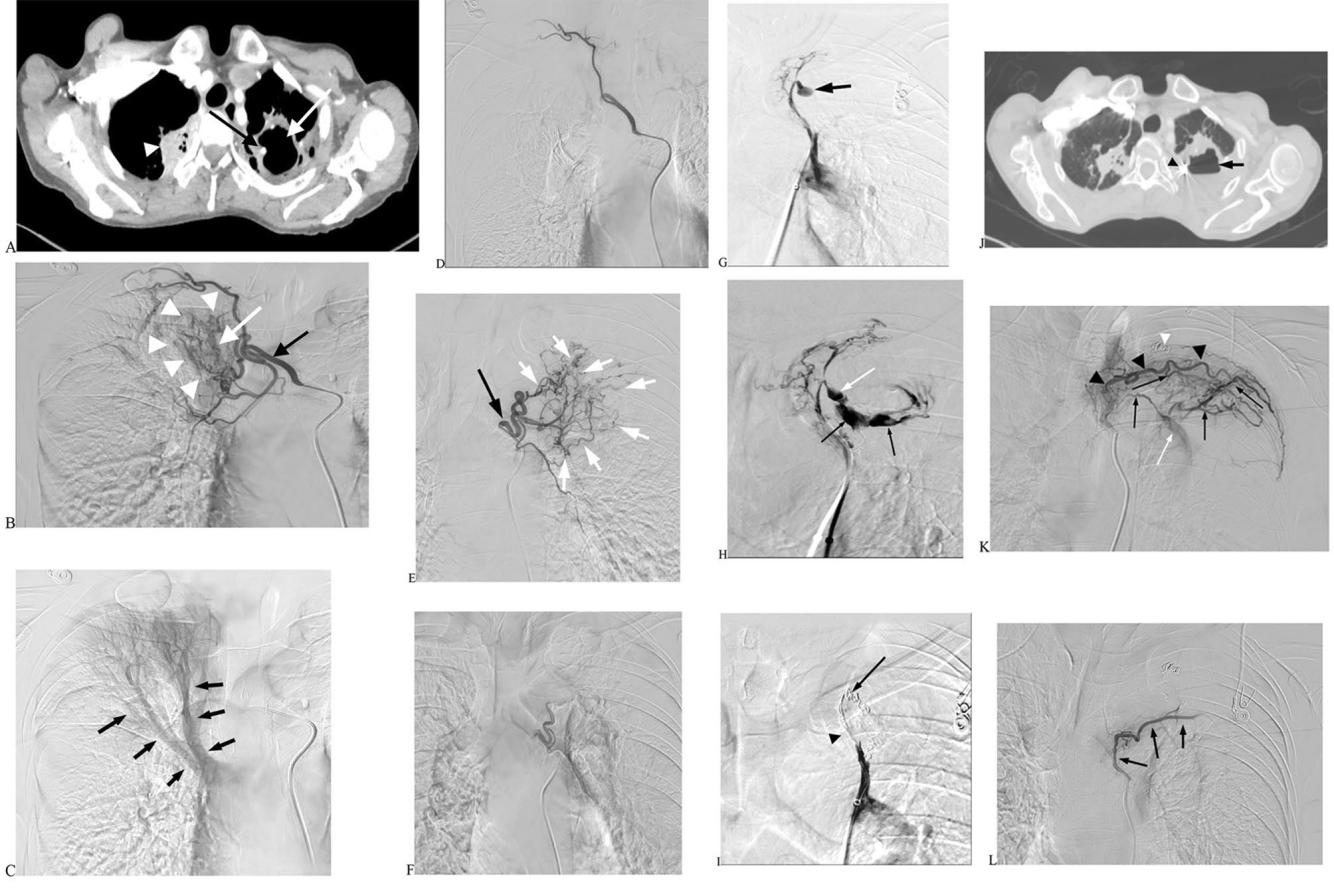
The patient with old pulmonary tuberculosis was hospitalized for 2 days with intermittent hemoptysis. The maximum hemoptysis volume in 24 h was approximately 700ml. **A**. Chest enhanced CT showing old lesions in the upper lobe of the right lung (white arrowhead). The left upper lobe cavity (white arrow) was formed, and PAP was observed in the cavity (black arrow). **B**. Right intercostal bronchial artery (black arrow) angiography shows obvious thickening, increased number, disorder, and structural abnormality of the bronchial artery (white arrowhead) and premature pulmonary artery branch (white arrow) in the upper lobe of the right lung. **C**. Right intercostal bronchial arteriography shows reverse flow in the late stage of the artery. The angiography also shows the pulmonary artery (black arrow) in the upper lobe of the right lung. **D**. After intercostal bronchial artery embolization with 350-560 μm PVA particles, the main intercostal artery remained intact. The bronchial artery and its branches disappeared completely, indicating complete embolization. **E**. Left bronchial artery (black arrow) angiography shows obvious thickening, increased number, disorder, and structural abnormality of the bronchial artery (white arrow). No obvious PA or PAP was observed. **F**. Left bronchial artery angiography again shows left BA branch vessels disappeared post BAE in 350-560 μm PVA particles. **G**. PAP showed on the left upper lobe of the lung by introducing a single curved catheter assisted by the long vascular sheath (black arrow) pulmonary angiography. **H**. PAP (white arrow) rupture, and contrast agent entering the cavity (black arrow) as observed in pulmonary angiography. **I**. Angiography after PAPE + PAE with micro coils, dense embolization with micro coils in parent artery (black arrowhead), and displacement of the micro coils at PAP rupture (black arrow). **J**. The patient was discharged from the hospital and admitted again with hemoptysis 13 months later. The amount of hemoptysis was about 100ml within 24 h. Enhanced chest CT examination showed that the cavity in the upper lobe of the left lung (black arrow) was smaller than before, and the shadow of the micro coils was visible within it (black arrowhead). **K**. Left intercostal artery (black arrowhead) angiography shows a large number of collateral vessels (black arrows) deviating into the lung, forming a large number of tortuous, thickened, malformed vessels, pulmonary artery fistula (white arrow), and micro coils shadow (white arrowhead). **L**. The presence of the main intercostal artery (black arrow) and the disappearance of collateral malformed vessels and pulmonary artery fistula were angiographed with 350-560 μm PVA embolization

The median follow-up time starting from the operation was 19.03 ± 1.59 (7–38) months. Ten patients had recurrent massive haemoptysis, and two patients died of suffocation (1 patient underwent PAPE, while the other underwent BAE + NBSAE + PAPE). However, eight patients did not have PAP recurrence after treatment (Fig. 3) (caused by new abnormal thickness, tortuosity, and expansion of BA or NBSA).

Complications were classified according to the Society of Interventional Radiology [9]. Although no major surgery-related complications were reported, 11 patients developed postoperative chest and back pain, discomfort, and other minor complications, which disappeared after symptomatic treatment with NBSAE + PAPE. The complications could have been mainly caused by protective embolization during NBSAE or ectopic embolization using a granulation embolization agent.

## Discussion

PAP induces a large amount of dark haemoptysis with no specific clinical manifestations in the intermittent stage [10, 11]. In this study, all 23 patients were admitted to the hospital due to massive haemoptysis, and a PAP was found during CTA before intravascular treatment. Most patients had an abnormally dilated tortuous BA and/or NBSA, but it could not be determined whether there was a definite BA and/or NBSA-PA fistula.

The upper lobe of the lungs is involved in recurrent tuberculosis [2, 12, 13, 14]. In this study, 2, 8, and 7 cases were located in the upper lobe of both lungs, upper lobe of the right lung, and upper lobe of the left lung, respectively (73.91% of the patients), which were consistent with the reported literature [11]. A PAP was detected in all the patients, of which two cases were reported in the main pulmonary lobar arteries (8.70%), and 21 cases were reported in the pulmonary lobar artery branch (91.30%). However, in the largest retrospective study conducted to date, a PAP was detected in 83% of patients, of which 63% of cases occurred in the pulmonary artery subsegment [11]. The discrepancy in this study could be because of the small sample size, or because most patients had pulmonary tuberculosis.

The source of bleeding can be first detected by CTA combined with PA and BA/NBSA angiography, providing information on the location, size, quantity, shape, and origin of PAP. In addition, CTA and angiography can detect dilation and tortuosity of the BA and NBSA [6, 8]. In this study, a PAP was detected by thoracic CTA in all the patients. An abnormally dilated tortuous BA or NBSA was also detected in most patients, but it could not be determined whether there were definite BA and/or NBSA-PA fistulas.

However, there is no consensus regarding the treatment guidelines for PAP. Conservative treatment can be conducted once the cause of PAP is identified to prevent rupture and bleeding. However, medication alone may not be effective in preventing an enlarged or ruptured PAP in patients with massive haemoptysis. Although various nonendovascular methods, such as image-guided percutaneous direct puncture embolization, PAP resection, and lobotomy, are applied in the treatment of patients who cannot receive endovascular treatment, they are associated with high mortality [15–18].

Endovascular treatment is minimally invasive, safe, and effective and is associated with low mortality. Endovascular therapy using different embolization materials has been explored. However, liquid embolization is poorly controlled and may result in ectopic embolism, pulmonary infarction, or catheter adhesion risk. Furthermore, vascular embolization can only embolize the inflow tract but cannot completely embolize the cavity and outflow tract, and thus, the blood may cause a countercurrent flow into the cavity. As a result [6, 8, 19], coil embolization, as a safe and effective method, is widely used for systemic aneurysms or pseudoaneurysms [20–22]. However, breathing or coughing is associated with a high risk of PAP rupture and position change during coil embolization. Therefore, coil embolization procedures should be carefully performed. The interlocking microcoil is very flexible and can be implanted via a microcatheter to ensure that the coil is very smooth in propulsion and can pass through very tortuous vessels. In addition, the forwards thrust force is small once the catheter has been inserted into the cavity, thus reducing the possibility of cavity rupture. Furthermore, the catheter tail has a mechanical torsion lock structure; thus, it can be recovered and released after adjustment if the release position is not ideal as long as the mechanical twist locking device of the tail is in the catheter [23]. In this study, interlock microcoils were used as PAP embolization material in all the patients.

PAP in the main pulmonary lobar arteries should be embolized with microcoils assisted by a bare stent. This is mainly because it is difficult for the coils to build a stable coil pack for wide-necked aneurysms. Furthermore, lung tissue contraction and expansion movements during breathing may cause coil migration for narrow-necked aneurysms. Although the covered stent can resolve the localized stenosis and isolate the aneurysm, the patency time of the covered stent is shorter than that of the bare stent, and the push delivery system of the covered stent is thicker, resulting in poor flexibility of the stent. This significantly increases the difficulty of release, and the tumour cavity cannot be filled. Microcoils combined with bare stents can more tightly embolize and make PA blood flow faster. The stent can resolve the PA stenosis caused by the inflammatory tissue around the aneurysm. Furthermore, the bare stent push device is thinner, more compliant than the covered stent, and easier to operate.

Embolization of a PAP in the pulmonary lobar artery branch vessels is relatively simple. In this study, an appropriately selected microcatheter was advanced as far as possible into the PA, and embolization was performed from the distal part of the pseudoaneurysm neck, resulting in the complete embolization of the entire aneurysm. This was conducted to prevent blood from returning to the aneurysm cavity and causing rupture and bleeding.

However, endovascular therapy for PAPs is relatively complex due to the presence of a BA and/or NBSA-PA fistula. As a result, PA angiography may not detect PAPs due to an A-P shunt but may be visible under BA and/or NBSA angiography [24]. As a result, simple embolization of the PAP and parent pulmonary artery may lead to recurrence. In this study, all patients underwent CTA of the BA and NBSA. A BA and/or NBSA-PA fistula was detected in 19 patients (82.61%) and was treated with embolization. In PAPE, the catheter was placed in the target BA and/or NBSA and then BAE and/or NBSAE was performed. PAPs be easily detected via pulmonary angiography before BAE and/or NBSAE.

A BA-PA and/or NBSA-PA fistula may be caused by pulmonary and pleural infection, pneumothorax, malignant tumour, or chest trauma leading to pulmonary hypertension [25–27]. Our results indicate that BA and/or NBSA-PA fistulas significantly increase PA pressure because the pressures and velocities of the BA and NBSA supplied by the systemic circulation are higher than those of the pulmonary artery [28, 29]. However, the fistula disappeared after embolization, and the PA pressure decreased. Furthermore, PAP coil embolization alone or pulmonary artery branch embolization did not significantly increase the PA pressure, suggesting that embolization is safe.

Although endovascular PAP therapy is less risky than surgery, it has similar risks to other systemic endovascular embolization procedures, including contrast-induced nephropathy, ectopic embolism, vascular dissections, thrombosis, and tissue infarction. In this study, 11 patients had postoperative chest and back pain, discomfort, and other minor complications, possibly caused by ectopic embolization during protective embolization or by a granulated embolization agent during NBSAE.

However, this study has some limitations. First, the sample size was small, thus limiting the generalizability of the conclusions. Second, a prospective, randomized, multicentre study is needed to clarify the effectiveness of PAPE for the treatment of massive haemoptysis.

## Conclusion

Endovascular methods are safe and effective for the treatment of massive haemoptysis caused by a PAP. BAE and/or NBSAE can effectively reduce pulmonary hypertension in patients with a BA-PA and/or NBSA-PA fistula.

## Data Availability

The data supporting the findings of this study are available from the corresponding author upon reasonable request.

## List of abbreviations

BA-PA: Bronchial artery-pulmonary artery
BA: Bronchial artery
BAE: Bronchial artery embolization
CTA: Computed tomography angiography
MDT: Multidisciplinary discussion
NBSA-PA: Nonbronchial systemic artery-pulmonary artery
NBSA: Nonbronchial systemic artery
NBSAE: Nonbronchial systemic artery embolization
PA: Pulmonary artery
PAE: Pulmonary artery embolization
PAP: Pulmonary pseudoaneurysm
